# Supplementary material for: Multicore fiber optic imaging reveals that astrocyte calcium activity in the mouse cerebral cortex is modulated by internal motivational state
Source: Nat Commun. 2024 Apr 8;15:3039. doi: 10.1038/s41467-024-47345-x (PMC11002016; doi:10.1038/s41467-024-47345-x)
Supplement: Supplementary file 1 — Supplementary Information [file 41467_2024_47345_MOESM1_ESM.pdf]

Multicore fiber optic imaging reveals that astrocyte calcium activity in the mouse cerebral cortex is modulated by internal motivational state

Supplementary Information

Supplementary Fig. 1: Representative field of view across a series of time points.

Supplementary Fig. 2: Distribution of behaviors in the untethered and tethered animals.

Supplementary Fig. 3: Imaging reflectance changes during behaviors.

Supplementary Fig. 4: Closer association between rise of astrocyte calcium and rearing offset.

Supplementary Fig. 5: Astrocyte events correlate with intrinsic cycle besides episodic transition.

Supplementary Table 1: Key resource table.

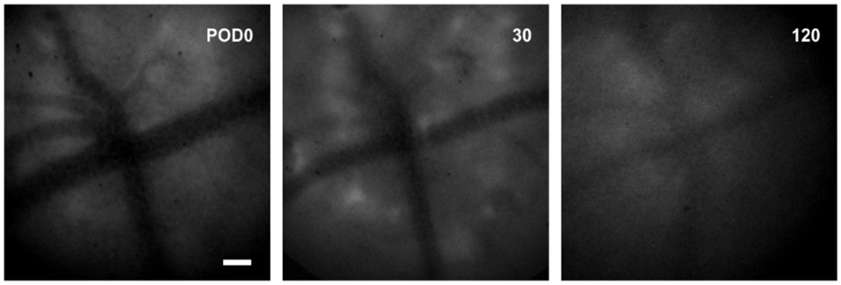

Supplementary Fig. 1: The representative field across a series of time points.

Surface blood vessels serve as fiducial landmarks to aid in positioning fibers to obtain the same field of view on post-operative day (POD) 0, 30 and 120. Scale bar is 50 μm. Source data are provided as a Source Data file.

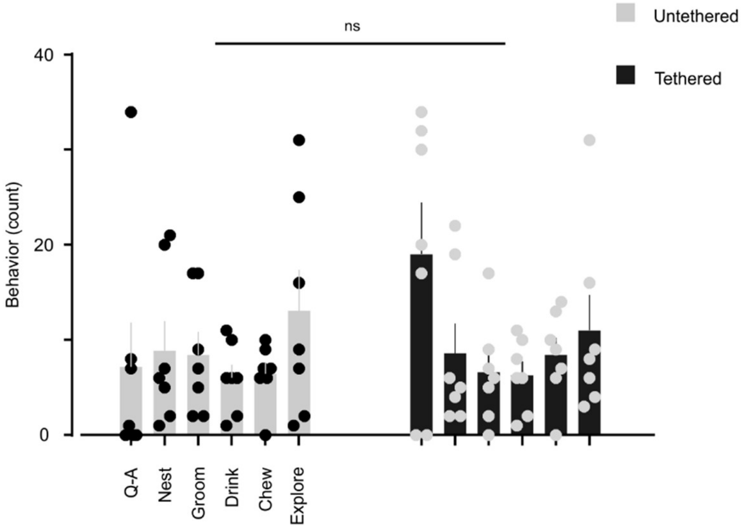

Supplementary Fig. 2: Distribution of behaviors in the untethered and tethered animals.

Phenotyping mouse behavior over one hour, showing that tethering to the optical fiber does not substantially change their pattern of spontaneous behavior (n = 7 animals for each group). Data are presented as mean ± s.e.m. Source data are provided as a Source Data file.

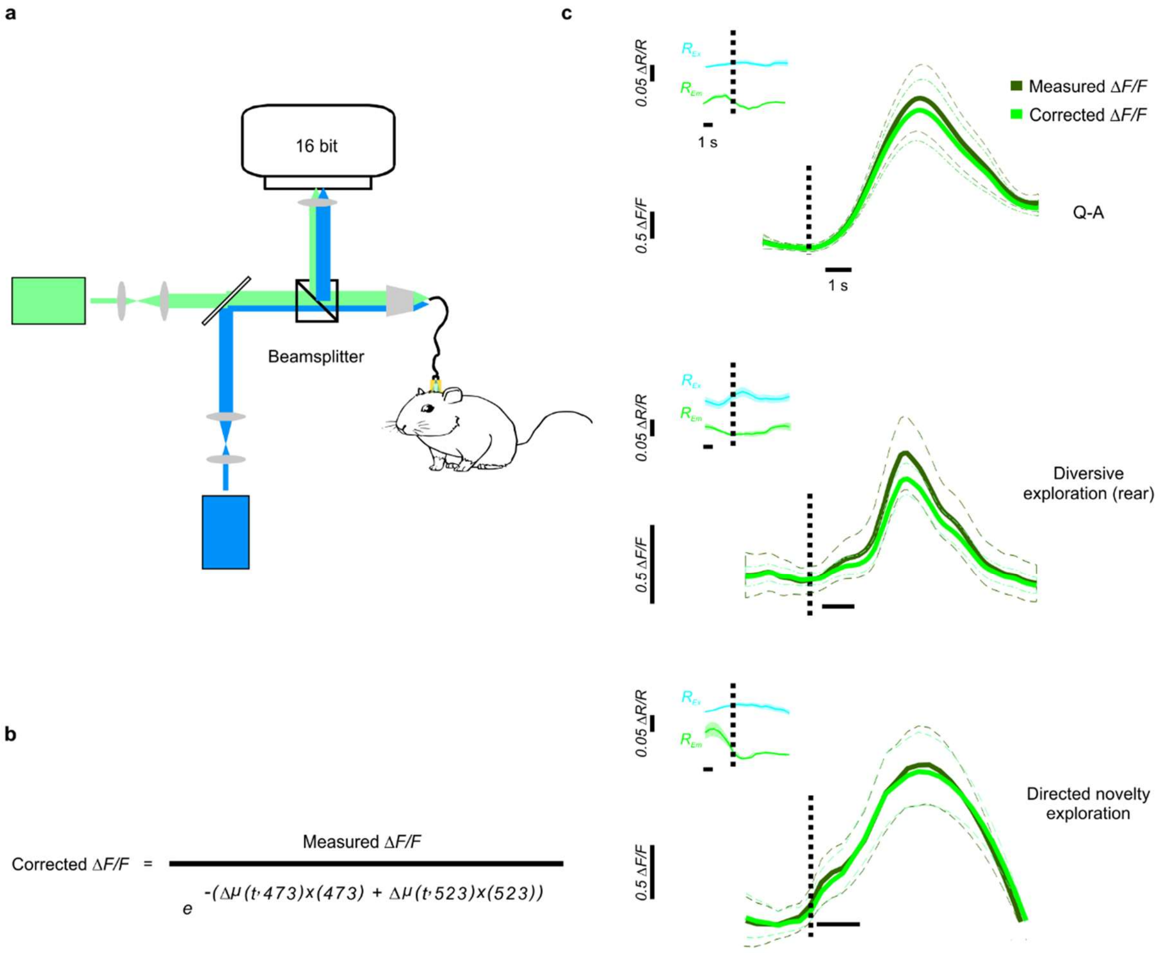

Supplementary Fig. 3: Imaging reflectance change during behaviors.

a, Freely moving reflectance imaging configuration recorded at 473 nm and 523 nm, the peak excitation/emission wavelengths used for GCaMP imaging. **b,** Formula derived from Ma et al. (2016) corrects GCaMP fluorescence signals for associated hemodynamic changes. The denominator, calculated from reflectance changes at 473 nm and 523 nm, is a negative exponential product of the time-varying absorption when the excitation and emission travel in the brain ($\mu_{(t, 473)}$ and $\mu_{(t, 523)}$) and their respective path lengths ($x_{(473)}$ and $x_{(523)}$). **c,** Reflectance changes had only a small effect on the amplitude and time course of GCaMP fluorescence signals in the major behaviors studied here. Data are presented as mean ± s.e.m.

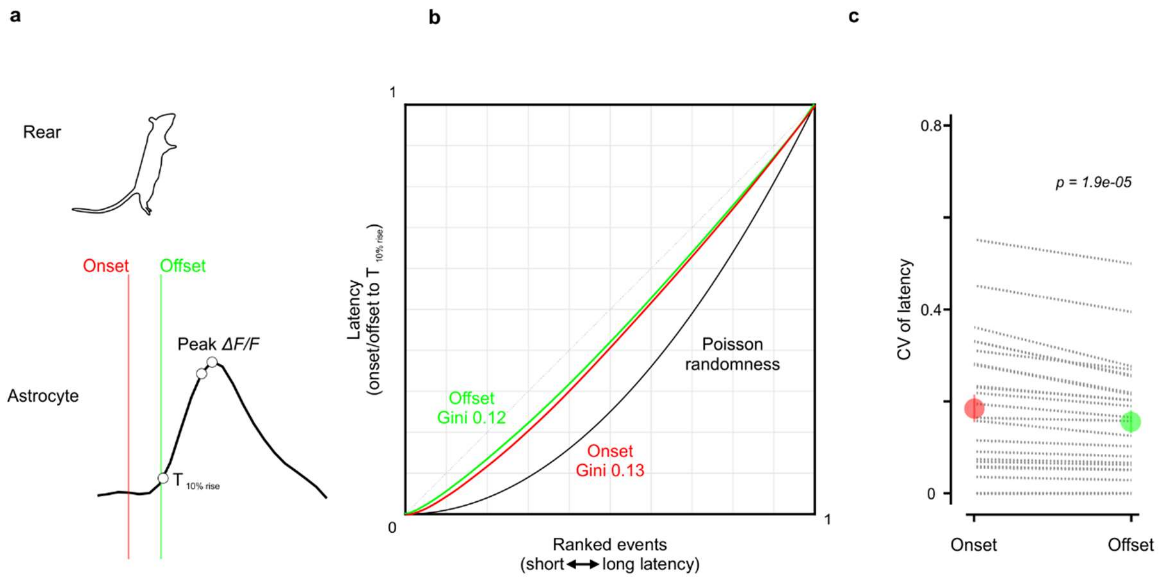

Supplementary Fig. 4: Association between rise of astrocyte calcium and rearing offset. **a,** Schematic diagram of the rearing onset (red), offset (green) and the time to 10% of peak calcium ($T_{10\% \text{ rise}}$). **b,** Lorenz plots for the latency of the $T_{10\% \text{ rise}}$ and the onset and offset times: Gini was 0.13 for onset latency distribution and 0.12 for offset. **c,** Coefficient of variation (CV) of latencies to the astrocyte 10% response across 25 animals was higher for the onset (red: 0.18 ± 0.03) than for the offset (green: 0.16 ± 0.03) (paired *t*-test). Data are presented as mean ± s.e.m. Source data are provided as a Source Data file

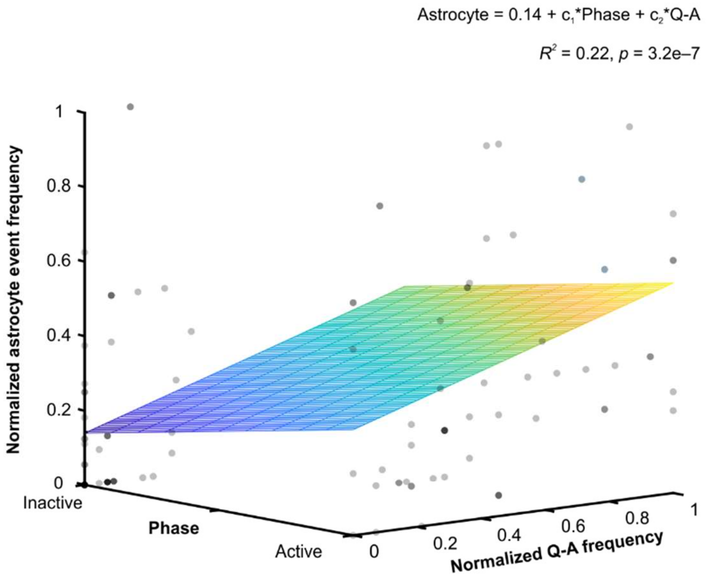

Supplementary Fig. 5: Astrocyte calcium events correlate with intrinsic cycle besides episodic transition.

Phase of activeness and Q-A transition are independently significantly correlation with astrocyte calcium activity (95% confidence interval: c_1 [0.03, 0.25], c_2 [0.08, 0.47]). The interaction between Phase*A was not included in the final analysis due to its statistical insignificance and inferior goodness of fit. Source data are provided as a Source Data file.

Supplementary Table 1: Key resources table.

Resource	Source	Identifier
**Antibodies**		
Normal Donkey Serum	Jackson ImmunoResearch	Cat# 017-000-121; RRID: AB_2337258
Chicken polyclonal anti-GFP	Aves Labs	Cat# GFP-1020; RRID: AB_10000240
Rabbit polyclonal anti-GFAP	Agilent Pathology Solutions	Cat# Z0334; RRID: AB_10013382
Mouse monoclonal anti-S100B	Sigma-Aldrich	Cat# S2532; RRID: AB_477499
Mouse monoclonal anti-NeuN	Sigma-Aldrich	Cat# MAB377; RRID: AB_2298772
Guinea pig polyclonal anti-Olig2	Bennett Novitch (UCLA)	Cat# Serum 252; RRID: AB_2715520
Alexa Fluor 488-conjugated AffiniPure $F(ab')_2$ Fragment Donkey anti-chicken IgY (IgG)(H+L)	Jackson ImmunoResearch	Cat# 703-546-155; RRID: AB_2340376
Donkey anti-Rabbit IgG (H+L) Cross-Adsorbed Secondary Antibody, DyLight 650	Thermo Fisher Scientific	Cat# SA5-10041; RRID: AB_2556621
Cy™3 AffiniPure Donkey Anti-Mouse IgG (H+L)	Jackson ImmunoResearch	Cat# 706-165-150; RRID: AB_2687868
Donkey Anti-Guinea pig IgG Antibody (H+L), Cy™3	Bioss	Cat# bs-0358D-Cy3; RRID: AB_10892938
**AAV**		
AAV.GFAP.iAChSnFR	Philip Borden, PhD<br>Loren Looger, PhD	Addgene plasmid #137955; RRID: Addgene 137995
AAV.GFAP.iAChSnFR-NULL	Philip Borden, PhD<br>Loren Looger, PhD	Addgene plasmid #137956; RRID: Addgene 137959

Chemicals, peptides, and recombinant proteins		
Dexmedetomidine hydrochloride	Tocris	Cat# 2749; CAS 145108-58-3
Prazosin hydrochloride	Tocris	Cat# 0623; CAS 19237-84-4
Atropine	Sigma-Aldrich	Cat# A0132; CAS 51-55-8
MTEP hydrochloride	Tocris	Cat# 2921; CAS 1186195-60-7
JNJ16259685	Tocris	Cat# 2333; CAS 409345-29-5
Methysergide maleate	Tocris	Cat# 1064, CAS 129-49-7
Tamoxifen	Sigma-Aldrich	Cat# T5648; CAS:10540-29-1
**Experimental models: organisms/strains**		
Tg(Slc1a3-cre/ERT)1Nat/J	Jeremy Nathans, MD PhD<br>Wang et al., 2012	IMSR_JAX:012586
Gt(ROSA)26Sor$^{tm1.1(CAG-EGFP)Fsh}$/Mmjax	Jackson Laboratory	MMRRC_032037-JAX
B6N;129-Gt(ROSA)26Sor$^{tm1(CAG-GCaMP3)Dbe}$/J	Dwight Bergles, PhD<br>Paukert et al., 2014	IMSR_JAX:028764
**Oligonucleotides**		
Primer: tcaatgggcgggggtcgtt (CMV-E-as)	Paukert et al., 2014	N/A
Primer: ctctgctgcctcctggcttct (ROSA26 s)	Paukert et al., 2014	N/A
Primer: cgaggcggatcacaagcaata (ROSA26-as)	Paukert et al., 2014	N/A
Primer: tgccacgaccaagtgacagcaatg (Cre-F)	Jeremy Nathans, MD PhD<br>Sigma-Aldrich	N/A
Primer: accagagacggaaatccatcgctc (Cre-R)	Jeremy Nathans, MD PhD<br>Sigma-Aldrich	N/A

Software and algorithms		
ZEN Blue/Black	Zeiss	RRID:SCR_013672 https://www.zeiss.com/microscopy/us/products/microscope-software/zen.html#downloads
Fiji	Schindelin et al., 2012	RRID:SCR_002285 http://fiji.sc
MATLAB 2019a	MathWorks	RRID:SCR_001622 https://www.mathworks.com/products/matlab.html
CorelDRAW Graphics Suite	Corel	RRID:SCR_014235 https://www.coreldraw.com/en/
SOLIDWORKS 2015	DS SolidWorks	RRID:SCR_024908 https://www.solidworks.com/product/solidworks-3d-cad
MiN1PIPE	Lu et al., 2018	https://www.mathworks.com/matlabcentral/fileexchange/72229-min1pipe
TV-L1 Image Denoising Algorithm	Lourakis, 2022 Chambolle and Pock, 2011	https://www.mathworks.com/matlabcentral/fileexchange/57604-tv-l1-image-denoising-algorithm
baseline_kde	Mitani and Komiyama, 2018	https://github.com/amitani/baseline_kde
CellReg	Sheintuch et al., 2017	https://www.mathworks.com/matlabcentral/fileexchange/69671-cellreg

iPeak	O'Haver, 2019	https://www.mathworks.com/matlabcentral/fileexchange/23850-ipeak
JAABA	Kabra et al., 2013	http://jaaba.sourceforge.net/
Vision-based system for automated mouse behavior recognition	Jhuang et al., 2010	https://cbmm.mit.edu/mouse-dataset
Code for data acquisition and analysis	Dwight Bergles, PhD	https://github.com/DEBLab01/NC2023
**Others**		
304F10125X012SL	TE connectivity	https://www.microgroup.com/product/304f10125x012sl/
304F10062X010SL	TE connectivity	https://www.microgroup.com/product/304f10062x010sl/
352140-A	Thorlabs	https://www.thorlabs.com/thorproduct.cfm?partnumber=352140-A
AC254-150-ML-A	Thorlabs	https://www.thorlabs.com/thorproduct.cfm?partnumber=AC254-150-A-ML
FF499-Di01-25x36	Semrock	https://www.edmundoptics.com/p/olympus-pln-20x-objective/29223/
FIGH-30-650S	Fujikura	https://www.fujikura.co.jp/eng/products/optical/opticalfibers/07/2050110_12902.html
BFS-PGE-13Y3C/M-C	FLIR	https://www.flir.com/products/blackfly-s-gige/?vertical=machine+vision&segment=iis
MF525-39	Thorlabs	https://www.thorlabs.com/thorproduct.cfm?partnumber=MF525-39
OBIS473L	Coherent	https://coherentinc.my.site.com/Coherent/1185052?cclcl=en_US

PLN 10×/0.25	Olympus	https://www.edmundoptics.com/p/olympus-pln-10x-objective/29222/
PLN 20×/0.4	Olympus	https://www.edmundoptics.com/p/olympus-pln-20x-objective/29223/
