# Supplementary material for: Multicore fiber optic imaging reveals that astrocyte calcium activity in the mouse cerebral cortex is modulated by internal motivational state
Source: Nat Commun. 2024 Apr 8;15:3039. doi: 10.1038/s41467-024-47345-x (PMC11002016; doi:10.1038/s41467-024-47345-x)
Supplement: Supplementary file 4 — Reporting Summary [file 41467_2024_47345_MOESM4_ESM.pdf]

[redacted]

Corresponding author(s): Yung-Tian A. Gau MD PhD, Jin U. Kang PhD, Dwight E. Bergles PhD

Last updated by author(s): 2024-03-11

Reporting Summary

Nature Portfolio wishes to improve the reproducibility of the work that we publish. This form provides structure for consistency and transparency in reporting. For further information on Nature Portfolio policies, see our Editorial Policies and the Editorial Policy Checklist.

Statistics

For all statistical analyses, confirm that the following items are present in the figure legend, table legend, main text, or Methods section.

n/a	Confirmed	
☐	☒	The exact sample size (*n*) for each experimental group/condition, given as a discrete number and unit of measurement
☐	☒	A statement on whether measurements were taken from distinct samples or whether the same sample was measured repeatedly
☐	☒	The statistical test(s) used AND whether they are one- or two-sided<br>*Only common tests should be described solely by name; describe more complex techniques in the Methods section.*
☐	☒	A description of all covariates tested
☐	☒	A description of any assumptions or corrections, such as tests of normality and adjustment for multiple comparisons
☐	☒	A full description of the statistical parameters including central tendency (e.g. means) or other basic estimates (e.g. regression coefficient) AND variation (e.g. standard deviation) or associated estimates of uncertainty (e.g. confidence intervals)
☐	☒	For null hypothesis testing, the test statistic (e.g. *F*, *t*, *r*) with confidence intervals, effect sizes, degrees of freedom and *P* value noted<br>*Give P values as exact values whenever suitable.*
☐	☒	For Bayesian analysis, information on the choice of priors and Markov chain Monte Carlo settings
☒	☐	For hierarchical and complex designs, identification of the appropriate level for tests and full reporting of outcomes
☒	☐	Estimates of effect sizes (e.g. Cohen's *d*, Pearson's *r*), indicating how they were calculated

Our web collection on statistics for biologists contains articles on many of the points above.

Software and code

Policy information about availability of computer code

Data collection	C# code for data collection is available at https://github.com/DEBLab01/NC2023.
Data analysis	MATLAB code for data analysis is available at https://github.com/DEBLab01/NC2023.

For manuscripts utilizing custom algorithms or software that are central to the research but not yet described in published literature, software must be made available to editors and reviewers. We strongly encourage code deposition in a community repository (e.g. GitHub). See the Nature Portfolio guidelines for submitting code & software for further information.

Data

Policy information about availability of data

All manuscripts must include a data availability statement. This statement should provide the following information, where applicable:

- Accession codes, unique identifiers, or web links for publicly available datasets
- A description of any restrictions on data availability
- For clinical datasets or third party data, please ensure that the statement adheres to our policy

The source data are provided in the Source Data file. Sample images are available in the Figshare repository at doi.org/10.6084/m9.figshare.23542803.v1
Larger sets of images are available from the lead contact upon request.

Research involving human participants, their data, or biological material

Policy information about studies with human participants or human data. See also policy information about sex, gender (identity/presentation), and sexual orientation and race, ethnicity and racism.

Reporting on sex and gender	N/A
Reporting on race, ethnicity, or other socially relevant groupings	N/A
Population characteristics	N/A
Recruitment	N/A
Ethics oversight	N/A

Note that full information on the approval of the study protocol must also be provided in the manuscript.

Field-specific reporting

Please select the one below that is the best fit for your research. If you are not sure, read the appropriate sections before making your selection.

☒ Life sciences ☐ Behavioural & social sciences ☐ Ecological, evolutionary & environmental sciences

For a reference copy of the document with all sections, see nature.com/documents/nr-reporting-summary-flat.pdf

Life sciences study design

All studies must disclose on these points even when the disclosure is negative.

Sample size	No statistical method was used to determine sample size. Sample size was chosen based on standards in the field, for example Armbruster, M., Naskar, S., Garcia, J. P., Sommer, M., Kim, E., Adam, Y., Haydon, P. G., Boyden, E. S., Cohen, A. E., & Dulla, C. G. (2022). Neuronal activity drives pathway-specific depolarization of peripheral astrocyte processes. Nature neuroscience, 25(5), 607–616. https://doi.org/10.1038/s41593-022-01049-x
Data exclusions	No data were excluded from the analysis.
Replication	Fluorescence light microscopy experiments were done in triplicate with similar results.<br>Multiple events are observed in each animal and multiple animals were used for individual in vivo experiments.
Randomization	Animals were selected and allocated into control vs experimental groups using random number generator.
Blinding	The persons performing experiment and analysis were unaware of the animal identity.

Reporting for specific materials, systems and methods

We require information from authors about some types of materials, experimental systems and methods used in many studies. Here, indicate whether each material, system or method listed is relevant to your study. If you are not sure if a list item applies to your research, read the appropriate section before selecting a response.

Materials & experimental systems

n/a	Involved in the study	
☐	☒	Antibodies
☒	☐	Eukaryotic cell lines
☒	☐	Palaeontology and archaeology
☐	☒	Animals and other organisms
☒	☐	Clinical data
☒	☐	Dual use research of concern
☒	☐	Plants

Methods

n/a	Involved in the study	
☒	☐	ChIP-seq
☒	☐	Flow cytometry
☒	☐	MRI-based neuroimaging

Antibodies

Antibodies used	Chicken polyclonal anti-GFP Aves Labs Cat# GFP-1020; RRID: AB_10000240<br>Rabbit polyclonal anti-GFAP Agilent Pathology Solutions Cat# Z0334; RRID: AB_10013382<br>Mouse monoclonal anti-S100B Sigma-Aldrich Cat# S2532; RRID: AB_477499<br>Mouse monoclonal anti-NeuN Sigma-Aldrich Cat# MAB377; RRID: AB_2298772<br>Guinea pig polyclonal anti-Olig2 Bennett Novitch (UCLA) Cat# Serum 252; RRID: AB_2715520<br>Alexa Fluor 488-conjugated AffiniPure F(ab')2 Fragment Donkey anti-chicken IgY (IgG)(H+L) Jackson ImmunoResearch Cat# 703-546-155; RRID: AB_2340376<br>Donkey anti-Rabbit IgG (H+L) Cross-Adsorbed Secondary Antibody, DyLight 650 Thermo Fisher Scientific Cat# SA5-10041; RRID: AB_2556621<br>Cy™3 AffiniPure Donkey Anti-Mouse IgG (H+L) Jackson ImmunoResearch Cat# 706-165-150; RRID: AB_2687868<br>Donkey Anti-Guinea pig IgG Antibody (H+L), Cy™3 Bioss Cat# bs-0358D-Cy3; RRID: AB_10892938
Validation	All primary antibodies used have been validated for immunohistochemistry or Immunofluorescence in mice. Details for individual antibodies are listed as below.<br><br>https://www.aveslabs.com/products/anti-green-fluorescent-protein-antibody-gfp#IHC<br>Calatayud-Baselga, I., Casares-Crespo, L., Franch-Ibáñez, C., Guijarro-Nuez, J., Sanz, P., & Mira, H. (2023). Autophagy drives the conversion of developmental neural stem cells to the adult quiescent state. Nature communications, 14(1), 7541. https://doi.org/10.1038/s41467-023-43222-1<br><br>https://www.agilent.com/en/product/immunohistochemistry/antibodies-controls/primary-antibodies/glial-fibrillary-acidic-protein-%28concentrate%29-76683#specifications<br>Günther, H. S., Henne, S., Oehlmann, J., Urban, J., Pleizier, D., Renevier, N., Lohr, C., & Wülfing, C. (2021). GFAP and desmin expression in lymphatic tissues leads to difficulties in distinguishing between glial and stromal cells. Scientific reports, 11(1), 13322. https://doi.org/10.1038/s41598-021-92364-z<br><br>https://www.sigmaaldrich.com/US/en/product/sigma/amab91038#product-documentation<br>Liu, Z., Chao, J., Wang, C., Sun, G., Roeth, D., Liu, W., Chen, X., Li, L., Tian, E., Feng, L., Davtyan, H., Blurton-Jones, M., Kalkum, M., & Shi, Y. (2023). Astrocytic response mediated by the CLU risk allele inhibits OPC proliferation and myelination in a human iPSC model. Cell reports, 42(8), 112841. https://doi.org/10.1016/j.celrep.2023.112841<br><br>https://www.sigmaaldrich.com/US/en/product/mm/mab377<br>Kellner, V., Kersbergen, C. J., Li, S., Babola, T. A., Saher, G., & Bergles, D. E. (2021). Dual metabotropic glutamate receptor signaling enables coordination of astrocyte and neuron activity in developing sensory domains. Neuron, 109(16), 2545–2555.e7. https://doi.org/10.1016/j.neuron.2021.06.010<br><br>Skaggs, K., Martin, D. M., & Novitch, B. G. (2011). Regulation of spinal interneuron development by the Olig-related protein Bhlhb5 and Notch signaling. Development (Cambridge, England), 138(15), 3199–3211. https://doi.org/10.1242/dev.057281

Animals and other research organisms

Policy information about studies involving animals; ARRIVE guidelines recommended for reporting animal research, and Sex and Gender in Research

Laboratory animals	We included in this paper mice of random sex, mixed B6N;129 background and between 20 to 24-weeks-old. The animals could assess water and food ad libitum in standard polycarbonate cages with enrichment. The housing facilities are maintained at 40–60% humidity, at a temperature of 20–25ºC and on a 12-hour light/dark cycle.<br>Generation of the following mouse lines have been previously published: Tg(Slc1a3-cre/ERT)1Nat/J , also known as GLAST-CreER (Wang et al., 2012); STOCK Gt(ROSA)26Sortm1.1(CAG-EGFP)Fsh/Mmjax, also known as RCE:loxP (Sousa et al., 2009), B6N;129-Gt(ROSA)26Sortm1(CAG-GCaMP3)Dbe/J, also known as Rosa26-lsl-GCaMP3 (Paukert et al., 2014). Corresponding RRIDs are listed in key resources table. We acquired these mice from the Jackson Laboratory and crossed the Cre recombinase-conditional EGFP (RCE:loxP) or GCaMP (Rosa26-lsl-GCaMP3) to the Cre-bearing (GLAST-CreER) mice. Offspring (GLAST-CreER; RCE:loxP or GLAST-CreER; Rosa26-lsl-GCaMP3 mice) were then exposed to tamoxifen to induce selective expression of EGFP or GCaMP in astrocytes.
Wild animals	No wild animals were used.
Reporting on sex	We included in this paper mice of random sex.
Field-collected samples	No wild animals were used.
Ethics oversight	All experiments and procedures were approved by the Johns Hopkins Institutional Care and Use Committee.

Note that full information on the approval of the study protocol must also be provided in the manuscript.
